# A 12-lead electrocardiogram database for arrhythmia research covering more than 10,000 patients

**DOI:** 10.1038/s41597-020-0386-x

**Published:** 2020-02-12

**Authors:** Jianwei Zheng, Jianming Zhang, Sidy Danioko, Hai Yao, Hangyuan Guo, Cyril Rakovski

**Affiliations:** 1 Chapman University, Orange, USA; 2 Shaoxing People’s Hospital (Shaoxing Hospital Zhejiang University School of Medicine), Shaoxing, China; 3 Zhejiang Cachet Jetboom Medical Devices CO.LTD, Hangzhou, China

**Keywords:** Atrial fibrillation, Biomedical engineering, Scientific data, Statistics, Physical examination

## Abstract

This newly inaugurated research database for 12-lead electrocardiogram signals was created under the auspices of Chapman University and Shaoxing People’s Hospital (Shaoxing Hospital Zhejiang University School of Medicine) and aims to enable the scientific community in conducting new studies on arrhythmia and other cardiovascular conditions. Certain types of arrhythmias, such as atrial fibrillation, have a pronounced negative impact on public health, quality of life, and medical expenditures. As a non-invasive test, long term ECG monitoring is a major and vital diagnostic tool for detecting these conditions. This practice, however, generates large amounts of data, the analysis of which requires considerable time and effort by human experts. Advancement of modern machine learning and statistical tools can be trained on high quality, large data to achieve exceptional levels of automated diagnostic accuracy. Thus, we collected and disseminated this novel database that contains 12-lead ECGs of 10,646 patients with a 500 Hz sampling rate that features 11 common rhythms and 67 additional cardiovascular conditions, all labeled by professional experts. The dataset consists of 10-second, 12-dimension ECGs and labels for rhythms and other conditions for each subject. The dataset can be used to design, compare, and fine-tune new and classical statistical and machine learning techniques in studies focused on arrhythmia and other cardiovascular conditions.

## Abstract (summary)

**Table:** 

Measurement(s)	cardiac arrhythmia
Technology Type(s)	12 lead electrocardiography • digital curation
Factor Type(s)	sex • experimental condition • age group
Sample Characteristic - Organism	Homo sapiens

Machine-accessible metadata file describing the reported data: 10.6084/m9.figshare.11698521

## Background & Summary

**Fig. 1:**
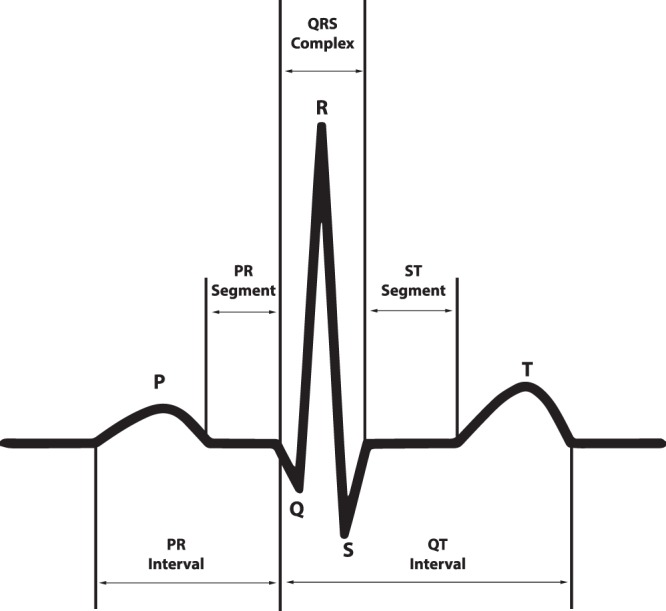
The ECG waveform and segments in lead II that presents a normal cardiac cycle.

An ECG is a graph of voltage with respect to time that reflects the electrical activities of cardiac muscle depolarization followed by repolarization during each heartbeat. The ECG graph of a normal beat (shown in Fig. 1) consists of a sequence of waves, a P-wave presenting the atrial depolarization process, a QRS complex denoting the ventricular depolarization process, and a T-wave representing the ventricular repolarization. Other portions of the signal include the PR, ST, and QT intervals. Arrhythmias represent a family of cardiac conditions characterized by irregularities in the rate or rhythm of heartbeats. There are several dozen such classes with various distinct manifestations such as excessively slow or fast heartbeats (sinus bradycardia (SB) and atrial tachycardia (AT)) and irregular rhythm with missing or distorted wave segments and intervals (premature ventricular contraction (PVC)). The most common and pernicious arrhythmia type is atrial fibrillation (AFIB). It is associated with a significant increase in the risk of severe cardiac dysfunction and stroke. Recent reports from the American Heart Association^1^ outlined that, in 2015, AFIB was the underlying cause of death for 23,862 people and was listed on 148,672 US death certificates. In 2010, the estimates of the prevalence of AFIB in the United States ranged from 2.7 million to 6.1 million. According to the same report, AFIB prevalence is expected to rise to 12.1 million in 2030. This alarming situation is not unique to the US. In fact, in Europe, the prevalence of AFIB in adults older than 55 years was estimated to be 8.8 million (95% CI, 6.5–12.3 million) and was projected to rise to 17.9 million by 2060 (95% CI, 13.6–23.7 million). The prevalence of AFIB in the Chinese population aged 35 years or older was 0.71%^2^. A significant contribution of this database is that it contains 3,889 subjects with AFIB rhythm.

According to the current screening and diagnostic practices, cardiologists or physicians review ECG data, establish the correct diagnosis, and begin implementing subsequent treatment plans such as medication regime and radiofrequency catheter ablation. However, the demand for high accuracy automatic heart condition diagnoses has recently increased sharply in parallel with the public health policy of implementing wider screening procedures and the adoption of ECG enabled wearable devices. Such classification methods require large size data that contain all prevalent types of conditions for algorithm training purposes.

There are several labeled, publicly available ECG databases such as the MIT-BIH arrhythmia database^3^, European ST-T database^4^, Creighton University ventricular tachycardia arrhythmia database, and St. Petersburg Institute of Cardiological Technics 12-lead arrhythmia database^5^. The American Heart Association (AHA) developed a database of arrhythmias and normal ECGs that contains 154 beat-by-beat annotated recordings, but it is not available for public use. These databases are either single lead or 12-lead ECG with sampling frequency less than 500 Hz and sample size smaller than 200. The sampling frequency is important in capturing certain vital cardiac conditions. For example, pacemaker stimulus outputs are generally shorter in duration by 0.5 ms, and therefore, they cannot be reliably detected by ordinary signal collection technique with sampling rates between 500 and 1000 Hz^6^. We compared the characteristics of the above-mentioned datasets and the one proposed in this paper (shown in Table 1). Our database contains the largest number of subjects, the highest sampling rate and the largest number of leads. Further, it also includes 11 heart rhythms and 56 types of cardiovascular conditions labeled by professional physicians. Additionally, the database includes basic ECG measurements such as QRS counts, atrial beat rate, ventricle beat rate, Q offset, and T offset.

**Table 1:** 7 ECG databases comparison.

Name	Subjects	Records (length)	Sampling rate	Age	Male, n(%)	Lead, n
MIT-BIH	47	48 (30 min)	360 Hz	23–89	25 (52.08)	2
EDB	79	90 (120 min)	250 Hz	30–84	70 (88.61)	2
AHA	N/A	154 (180 min)	250 Hz	N/A	N/A	2
CU	35	35 (8 min)	250 Hz	N/A	N/A	2
NSD	2	12 (30 min)	360 Hz	51–69	1 (50)	2
St Petersburg DB	32	75 (30 min)	257 Hz	18–80	17 (53.13)	12
Proposed one	10646	10646 (10 second)	500 Hz	4–98	5956 (55.95)	12

## Methods

### Participants and digitization parameters

Our data consists of 10,646 patient ECGs including 5,956 males and 4,690 females. Among those patients, 17% had normal sinus rhythm and 83% had at least one abnormality. The age groups with the highest prevalence were 51–60, 61–70 and 71–80 years representing 19.82%, 24.38%, and 16.9%, respectively. A detailed description of the enrolled participants’ baseline characteristics and rhythm frequency distribution is presented in Table 2. The number of volts per A/D bit is 4.88, and A/D converter had 32-bit resolution. The amplitude unit was microvolt. The upper limit was 32,767, and the lower limit was −32,768. The institutional review board of Shaoxing People’s Hospital approved this study, granted the waiver application to obtain informed consent, and allowed the data to be shared publicly after de-identification.

**Table 2:** Rhythm information and baseline characteristics of participants.

Acronym Name	Full Name	Frequency, n(%)	Age, Mean ± SD	Male, n(%)
SB	Sinus Bradycardia	3,889 (36.53)	58.34 ± 13.95	2,481 (58.48%)
SR	Sinus Rhythm	1,826 (17.15)	54.35 ± 16.33	1,024 (56.08%)
AFIB	Atrial Fibrillation	1,780 (16.72)	73.36 ± 11.14	1,041 (58.48%)
ST	Sinus Tachycardia	1,568 (14.73)	54.57 ± 21.06	799 (50.96%)
AF	Atrial Flutter	445 (4.18)	71.07 ± 13.5	257 (57.75%)
SI	Sinus Irregularity	399 (3.75)	34.75 ± 23.03	223 (55.89%)
SVT	Supraventricular Tachycardia	587 (5.51)	55.62 ± 18.53	308 (52.47%)
AT	Atrial Tachycardia	121 (1.14)	65.72 ± 19.3	64 (52.89%)
AVNRT	Atrioventricular Node Reentrant Tachycardia	16 (0.15)	57.88 ± 17.34	12 (75%)
AVRT	Atrioventricular Reentrant Tachycardia	8 (0.07)	57.5 ± 16.84	5 (62.5%)
SAAWR	Sinus Atrium to Atrial Wandering Rhythm	7 (0.07)	51.14 ± 31.83	6 (85.71%)
All	All	10,646 (100)	51.19 ± 18.03	5,956 (55.95%)

### Data acquisition

The data were acquired in four stages. First, each subject underwent a 12-lead resting ECG test that was taken over a period of 10 seconds. The data were stored into the GE MUSE ECG system. Second, a licensed physician labeled the rhythm and other cardiac conditions. Another licensed physician performed a secondary validation. If there was a disagreement, a senior physician intervened and made a final decision. There are labels of each subject’s rhythm and other conditions such as PVC, right bundle branch block (RBBB), left bundle branch block (LBBB), and atrial premature beat (APB). These additional conditions were applied to the entire sample rather than to specified beats in the 10-second reading. The final diagnoses were stored in the MUSE ECG system as well. Third, ECG data and diagnostic information were exported from the GE MUSE system to XML files that were encoded with specific naming conversion defined by General Electric (GE). Finally, we developed a converting tool to extract ECG data and diagnostic information from the XML file and transfer them to CSV format. In doing so, we referred to the work of Maarten J.B. van Ettinger (https://sourceforge.net/projects/ecgtoolkit-cs/).

### Data denoising method

**Fig. 2:**
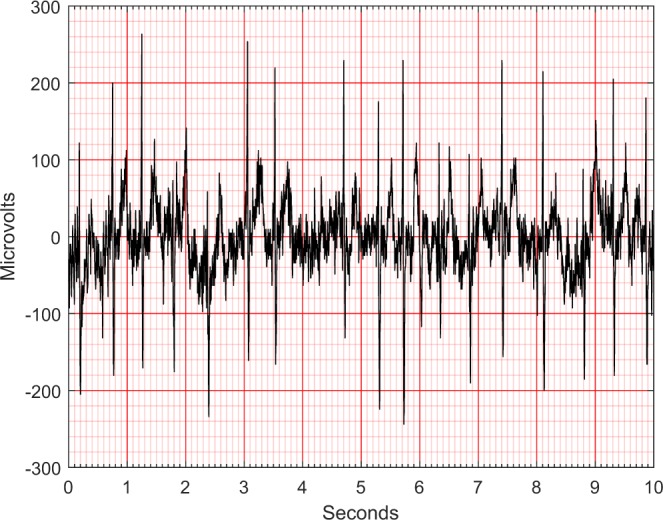
An ECG containing both low and high frequency noise.

**Fig. 3:**
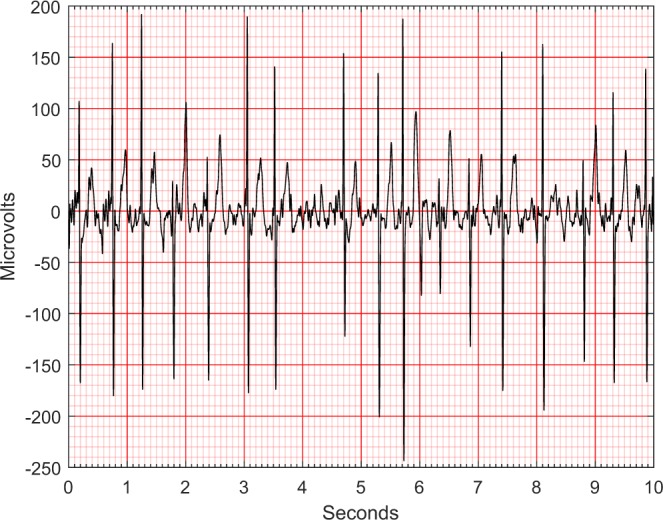
An ECG after noise reduction.

**Fig. 4:**
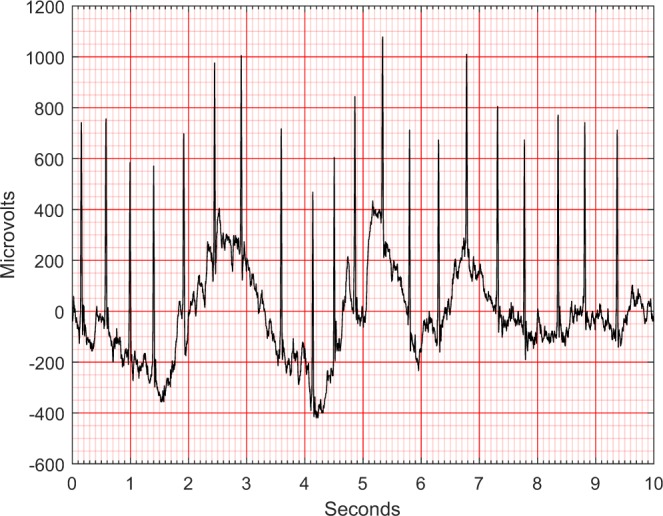
An ECG containing baseline wandering.

**Fig. 5:**
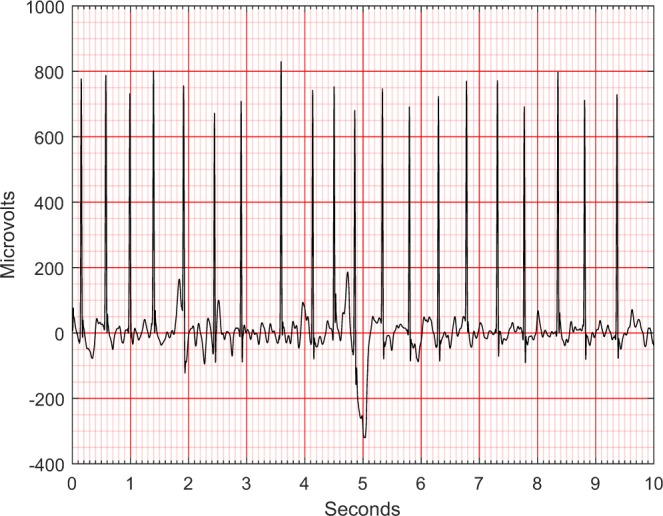
An ECG after removing baseline wandering.

In this study, the noise contamination sources in the ECG data were due to power line interference, electrode contact noise, motion artifacts, muscle contraction, baseline wandering, and random noise. As well known, the presence of noise can be a remarkable obstacle to any statistical analysis. Thus, we proposed and implemented a sequential noise reduction approach to process raw ECG data. Since the frequency range of normal ECG is from 0.5 Hz to 50 Hz, the Butterworth low pass filter was used to remove the signal with a frequency above 50 Hz. Then, LOESS smoother was utilized to clear the effects of baseline wandering. Lastly, the Non Local Means (NLM) technique was used to handle the remaining noise. One ECG sample containing both low and high frequency noise was presented in Fig. 2, whereas the noise reduction performance was displayed in Fig. 3. Another ECG sample contaminated by baseline wandering is shown in Fig. 4, and the effectiveness of LOESS smoother was demonstrated in Fig. 5. To get a full understanding of the techniques and the scheme that was adopted, please refer to the source code in the Code Availability section.

#### Butterworth low pass filter

Butterworth is a filter that was first introduced in 1930 by the British engineer and physicist Stephen Butterworth^7^. Its merit comes from the fact that its frequency response is as flat as possible in the passband. We set up parameters of the filter as follows: passband to 50 Hz, stopband to 60 Hz, no more than 1.0 dB of passband ripple and at least 2.5 dB of attenuation in the stopband. The filtering would not only change the amplitude but also shift the phase that is disadvantageous for subsequent analyses. Thus, we performed filtering in both forward and reverse directions to compensate for this phase-shifting.

#### LOESS curve fitting

The local polynomial regression smoother (LOESS)^8,9^ was used to remove baseline wandering. The smoother was fitted using weighted least squares where the weight function gives the most weight to the data points nearest the point of estimation and the least weight to the data points that farthest away. We used a robust version of LOESS that assigns zero weight to data outside six mean absolute deviations. We subtracted the LOESS estimated trend to clear the effect of baseline wandering.

#### Non local means(NLM)

The NLM was also used for residual noise reduction. This algorithm was first introduced to smooth the repeated structures in digital images^10^. Later, this idea was applied to ECG data denoising^11^, and further developed and combined with Empirical Mode Decomposition^12^. For a certain length of univariate time series data, NLM reconstructs every data point *S*(*i*) through weighted averaging of all data points *D*(*i*) in the original sequence, where *i* and *j* are indices of location. The weights *w*(*i*, *j*) are determined by a similarity measure between *D*(*i* + *δ*) and *D*(*i* + *δ*), *δ* ∈ Δ.

1 $$S(i)=\frac{1}{Z(i)}\sum _{j\in N(i)}\,w(i,j)D(j)$$

where

2 $$Z(i)=\sum _{j}\,w(i,j)$$

and

3 $$w(i,j)=exp\left(-\frac{\sum _{\delta \in \Delta }\,{[D(i+\delta )-D(j+\delta )]}^{2}}{2{L}_{\Delta }{\lambda }^{2}}\right)$$

where *λ* is a smoothness control parameter, and Δ represents a local patch of samples containing *L*_Δ_ samples. Thus, at each point, the NLM smoothing borrows information from all points that have similar patterns within the search range *N*(*i*). The similarity measure determines how many periods will be included and averaged. We used a Gaussian kernel as a weight function in the smoothing step of our analysis.

## Data Records

Data presented in this work consist of four parts: raw ECG data, denoised ECG data, diagnoses file, and attributes dictionary file. These files are available online at figshare^13^. For each subject, the raw ECG data were saved as a single CSV file, and denoised ECG data were saved under the same name CSV file, but in a different file folder. Also, each CSV file mentioned above contains 5000 rows and 12 columns with header names presenting the ECG lead. These CSV files are named by unique IDs. These IDs were also saved in the diagnostics file with attributes name *FileName*. The diagnoses file contains all the diagnoses information for each subject including filename, rhythm, other conditions, patient age, gender, and other ECG summary attributes (acquired from GE MUSE system). Table 3 displays detailed information for each attribute. The attribute dictionary file explains the acronym names of other cardiac conditions (shown in Online-only Table 1).

**Table 3:** Attributes in diagnosis file.

Attributes	Type	Value Range	Description
FileName	String		ECG data file name (unique ID)
Rhythm	String		Rhythm Label
Beat	String		Other conditions Label
PatientAge	Numeric	0–999	Age
Gender	String	MALE/FEMAL	Gender
VentricularRate	Numeric	0–999	Ventricular rate in BPM
AtrialRate	Numeric	0–999	Atrial rate in BPM
QRSDuration	Numeric	0–999	QRS duration in msec
QTInterval	Numeric	0–999	QT interval in msec
QTCorrected	Numeric	0–999	Corrected QT interval in msec
RAxis	Numeric	−179~180	R axis
TAxis	Numeric	−179~181	T axis
QRSCount	Numeric	0–254	QRS count
QOnset	Numeric	16 Bit Unsigned	Q onset (In samples)
QOffset	Numeric	17 Bit Unsigned	Q offset (In samples)
TOffset	Numeric	18 Bit Unsigned	T offset (In samples)

## Technical Validation

In this study, various technical approaches were employed to validate the reliability and quality of the ECG data. A detailed description of these validation methods was presented blow.

### ECG measurement validation

According to the standard ECG measurement mechanism, two constraints must be satisfied: first, the voltage value of lead II should always be equal to the sum of voltage values of lead I and lead III; second, the sum of voltage values of lead aVR, aVL, and aVF should be equal to zero. It is well known that the right hand electrode and left hand electrode could have their positions switched by operators without a change on corresponding ECG data. Moreover, some of the electrodes could slip off during the test resulting in ECGs displaying a straight line. We created an automatic error-checking algorithm that detects the presence of these undesirable cases and excluded such ECG records from the database.

### classification for validation

**Fig. 6:**
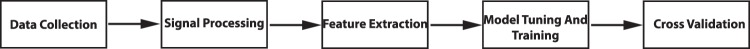
The common process of ECG analysis.

We implemented several arrhythmia classification algorithms on our data. The extreme gradient boosting tree^14^ attained the highest overall *F*_1_ score of 0.97. Detailed results were presented in Table 4. The high classification accuracy validates both the quality of the ECG data and the reliability of the arrhythmia condition labels. The pipeline of the proposed classification scheme was presented in Fig. 6.

**Table 4:** Performance report of gradient boosting tree model.

Rhythm group	F1-score	Precision	Recall
AFIB	0.941	0.938	0.944
GSVT	0.949	0.953	0.944
SB	0.993	0.990	0.996
SR	0.977	0.982	0.972
macro avg	0.965	0.966	0.964
micro avg	0.970	0.970	0.970
weighted avg	0.970	0.971	0.970

Since some rare rhythms have less than 10 samples as shown in Table 2, following a suggestion from cardiologists, we have hierarchically merged several rare cases to upper-level arrhythmia types. Thus, 11 rhythms were merged into 4 groups (SB, AFIB, GSVT, SR) shown in Table 5, SB only included sinus bradycardia, AFIB consisted of atrial fibrillation and atrial flutter (AF), GSVT contained supraventricular tachycardia, atrial tachycardia, atrioventricular node reentrant tachycardia, atrioventricular reentrant tachycardia and sinus atrium to atrial wandering rhythm, and SR included sinus rhythm and sinus irregularity. Referring to the guidelines^15–17^ that recommend AFIB and AF often coexist, any ECG with a rhythm of AFIB or AF was classified into AFIB group. Merging sinus rhythm and sinus irregularity to SR group helps to distinguish such a combination from the GSVT group, and sinus irregularity can be easily separated from sinus rhythm later by one single criterion, RR interval variation. Supraventricular tachycardia actually is a general term used in the daily ECG screening. For example, if the cardiologists cannot confirm atrial tachycardia or atrioventricular node reentrant tachycardia purely by ECG, they will give the general name supraventricular tachycardia. Therefore, the practice of merging all tachycardia originating from supraventricular locations to GSVT group was adopted in this work. After re-grouping labels of the dataset, these new aggregated classes can significantly contribute to the training of optimal classification approaches.

**Table 5:** The quantity of data after merged classes.

Merged from	Merged to	Total	Training data size (80%)	Testing data size (20%)
AFIB, AF	AFIB	3,889	3,111	778
SVT, AT, SAAWR, ST, AVNRT, AVRT	GSVT	2,307	1,846	461
SB	SB	2,225	1,780	455
SR, SI	SR	2,225	1,780	455
All	All	10,646	8,517	2,129

We designed a novel and interpretable feature extraction method. We added age and gender as features due to their importance in almost all medical data analyses. Features extracted from lead II include ventricular rate in beats per minute (BPM), atrial rate in BPM, QRS duration in millisecond, QT interval in millisecond, R axis, T axis, QRS count, Q onset, Q offset, mean of RR interval, Variance of RR interval, RR interval count. Features extracted from 12 leads contain mean and variance of height, width, prominence for QRS complex, non-QRS complex, and valleys. Peaks and valleys here represent the local maxima and minima. The prominence of a peak or a valley measures how much the peak or valley stands out due to its intrinsic height and its location relative to neighbor peaks or valleys. Thus, the prominence was defined as the vertical distance between the peak point and its lowest contour line. The peaks and valleys were assigned to three subsets, QRS complex, non-QRS peaks, and Valleys. In total, we created 230 features that were used in the extreme gradient boosting tree classification model described above. The *F*_1_ score of 0.97 is the average score from 10-fold cross-validation with 20% testing data. For each group, the sample sizes of training and testing datasets are presented in Table 5.

### Evaluation protocol for classification

For heartbeat classification evaluation, the ANSI/AAMI EC57 (R2012) gives a protocol and a database, the MIT-BIH arrhythmia database. Referring to the above industrial standard and the guidance from AHA, ACC, and HRS^6^, we proposed a five-step workflow for future study of rhythm classification.

1. Label selection:
  The available arrhythmia classification studies listed in^18^ classified heartbeats across all patients. In contrast, in this database, we used a clinically important rhythm classification that aggregates information from all beats into a single label. All rhythm labels are shown in Table 2. These rhythms can be combined according to different measures of similarity, as we demonstrated in the Classification for Validation section to increase sample size and address specific research questions.
2. Processing:
  We recommended a low-frequency filter to cut off 0.67 Hz or below with zero phase distortion, and a high-frequency filter with 50 Hz cutoff frequency. Using the raw ECG signal is also an option for classification scheme.
3. Feature extraction and selection:
  An interpretable feature extraction method is recommended. Using such a feature selection method, one can analyze feature importance and connection with physiological processes. Therefore, uninterpretable feature selection methods such as principal components analysis and neural networks are less desirable.
4. Classification:
  We encourage implementation and comparison of several competing classification schemes that include super-parameter tuning. The classification results need to report average performance accuracy using 10-fold validation.
5. Evaluation:
  *F*_1_-score, Overall Accuracy, Confusion Matrix, Precision (Positive Predictivity), and Recall (Sensitivity) are recommended to report classification performance.
  4 $${F}_{1}=2\ast \frac{Precision\;\ast \;Recall}{Precision+Recall}$$
  5 $$Overall\,Accuracy=\frac{True\,Positive+True\,Negative}{Total\,Population}$$
  6 $$Precision=\frac{True\,Positive}{True\,Positive+False\,Positive}$$
  7 $$Recall=\frac{True\,Positive}{True\,Positive+False\,Negative}$$

## Usage Notes

To get a better understanding of our approach, refer to a diagram shown in Fig. 6. In the data collection stage, we recommend the C# ECG Toolkit that is an open-source software to convert, view and print electrocardiograms (https://sourceforge.net/projects/ecgtoolkit-cs/). We suggest the use of Matlab or Python to carry out the denoising step of the analysis (see the Code Availability section). In the feature extraction step, BioSPPy (https://github.com/PIA-Group/BioSPPy/) is recommended to extract general ECG summary features such as QRS count, R wave location, etc. As for machine learning packages, we suggest scikit-learn^19^, and TensorFlow (https://www.tensorflow.org/) for deep learning model building.

## Data Availability

The source code of the converter tool that transfers ECG data files from XML format to CSV format can be found at https://github.com/zheng120/ECGConverter, which contains binary executable files, source code, and a user manual. Both the MATLAB (https://www.mathworks.com/) and Python version programs for ECG noise reduction are available at https://github.com/zheng120/ECGDenoisingTool.

## Online-only Table

**Online-only Table 1:** .

Acronym Name	Full Name
1AVB	1 degree atrioventricular block
2AVB	2 degree atrioventricular block
2AVB1	2 degree atrioventricular block(Type one)
2AVB2	2 degree atrioventricular block(Type two)
3AVB	3 degree atrioventricular block
ABI	atrial bigeminy
ALS	Axis left shift
APB	atrial premature beats
AQW	abnormal Q wave
ARS	Axis right shift
AVB	atrioventricular block
CCR	countercolockwise rotation
CR	colockwise rotation
ERV	Early repolarization of the ventricles
FQRS	fQRS Wave
IDC	Interior differences conduction
IVB	Intraventricular block
JEB	junctional escape beat
JPS	J point shift
JPT	junctional premature beat
LBBB	left bundle branch block
LBBBB	left back bundle branch block
LFBBB	left front bundle branch block
LRRI	Long RR interval
LVH	left ventricle hypertrophy
LVHV	left ventricle high voltage
LVQRSAL	lower voltage QRS in all lead
LVQRSCL	lower voltage QRS in chest lead
LVQRSLL	lower voltage QRS in limb lead
MI	myocardial infarction
MIBW	myocardial infraction in back wall
MIFW	Myocardial infgraction in the front wall
MILW	Myocardial infraction in the lower wall
MISW	Myocardial infraction in the side wall
PRIE	PR interval extension
PWC	P wave Change
QTIE	QT interval extension
RAH	right atrial hypertrophy
RAHV	right atrial high voltage
RBBB	right bundle branch block
RVH	right ventricle hypertrophy
STDD	ST drop down
STE	ST extension
STTC	ST-T Change
STTU	ST tilt up
TWC	T wave Change
TWO	T wave opposite
UW	U wave
VB	ventricular bigeminy
VEB	ventricular escape beat
VFW	ventricular fusion wave
VPB	ventricular premature beat
VPE	ventricular preexcitation
VET	ventricular escape trigeminy
WAVN	Wandering in the atrioventricalualr node
WPW	WPW

## References

[1] Benjamin J (2018). Heart Disease and Stroke Statistics-2018 Update: A Report From the American Heart Association. Circulation.

[2] Wang Z (2018). The Disease Burden of Atrial Fibrillation in China from a National Cross-sectional Survey. Am. J. Cardiol..

[3] Moody G, Mark R (2001). The impact of the MIT-BIH Arrhythmia Database. IEEE Eng. Med. Biol. Mag..

[4] Taddei A (1992). The European ST-T Database: standard for evaluating systems for the analysis of ST-T changes in ambulatory electrocardiography. Eur. Heart J..

[5] Goldberger A (2000). PhysioBank, PhysioToolkit, and PhysioNet. Circulation.

[6] Kligfield P (2007). Recommendations for the Standardization and Interpretation of the Electrocardiogram. Circulation.

[7] Butterworth S (1930). On the Theory of Filter Amplifiers. Wirel. Eng..

[8] Cleveland S (1979). Robust Locally Weighted Regression and Smoothing Scatterplots. JASA..

[9] Cleveland S, Devlin J (1988). Locally Weighted Regression: An Approach to Regression Analysis by Local Fitting. JASA..

[10] Buades A, Coll B, Morel J (2005). A Review of Image Denoising Algorithms, with A New One. SIMUL..

[11] Tracey H, Miller L (2012). Nonlocal Means Denoising of ECG Signals. IEEE Trans. Biomed. Eng..

[12] Tian X (2016). Electrocardiogram Signal Denoising Using Extreme-Point Symmetric Mode Decomposition and Nonlocal Means. Sens..

[13] Zheng J (2019). figshare.

[14] Chen, T. & Guestrin, C. XGBoost: A Scalable Tree Boosting System. *arXiv:1603.02754* (2016).

[15] January C (2014). 2014 AHA/ACC/HRS guideline for the management of patients with atrial fibrillation. JACC..

[16] Page R (2016). 2015 ACC/AHA/HRS guideline for the management of adult patients with supraventricular tachycardia. JACC..

[17] Kirchhof P (2016). 2016 ESC Guidelines for the management of atrial fibrillation developed in collaboration with EACTS. Eur. Heart J..

[18] Eduardo J, Schwartz R, Guillermo C, Menotti D (2016). ECG-based heartbeat classification for arrhythmia detection: A survey. Comput. Methods Prog. Biomed..

[19] Pedregosa F (2011). Scikit-learn: Machine Learning in Python. JMLR..

